# Identification of Surface Water Quality along the Coast of Sanya, South China Sea

**DOI:** 10.1371/journal.pone.0123515

**Published:** 2015-04-20

**Authors:** Zhen-Zhen Wu, Zhi-Wei Che, You-Shao Wang, Jun-De Dong, Mei-Lin Wu

**Affiliations:** 1 State Key Laboratory of Tropical Oceanography and Key Laboratory of Marine Bio-resources Sustainable Utilization, South China Sea Institute of Oceanology, Chinese Academy of Sciences, Guangzhou, China; 2 Haikou Marine Environmental Monitoring Central Station, State Oceanic Administration, Haikou, China; 3 Tropical Marine Biological Research Station in Hainan, Chinese Academy of Sciences, Sanya, China; CAS, CHINA

## Abstract

Principal component analysis (PCA) and cluster analysis (CA) are utilized to identify the effects caused by human activities on water quality along the coast of Sanya, South China Sea. PCA and CA identify the seasonality of water quality (dry and wet seasons) and polluted status (polluted area). The seasonality of water quality is related to climate change and Southeast monsoons. Spatial pattern is mainly related to anthropogenic activities (especially land input of pollutions). PCA reveals the characteristics underlying the generation of coastal water quality. The temporal and spatial variation of the trophic status along the coast of Sanya is governed by hydrodynamics and human activities. The results provide a novel typological understanding of seasonal trophic status in a shallow, tropical, open marine bay.

## Introduction

Nutrients entering oceans from surface runoff water of agricultural lands and urban areas have become a major environmental concern around the world [1]. Nutrient fluxes through these routes have been increased by human activity. In addition, the N:P:Si ratios of these inputs have been perturbed, and many coastal management practices exacerbate these perturbations [2]. Eutrophication and environmental pollution have obviously occurred in many coastal areas, especially in estuaries and coastal bays with dense human populations in their watersheds [3–7]. Eutrophication of aquatic environments stimulates primary production and leads to deleterious impacts on the structure and function of ecosystems, including the proliferation of harmful algal blooms[8]. Under anthropogenic stresses, deterioration of coastal ecosystems appears to be accelerating, but there is a paucity of knowledge on how complex aquatic communities are being altered in structure and function [9]. On the other hand, estuarine and coastal (jointly termed coastal) ecosystems are also affected by climatic change and associated perturbations, including droughts, hurricanes, and floods. Thus, understanding how anthropogenically induced change and nature changes (monsoon driven upwelling and mixing) affect estuarine and coastal ecosystem biodiversity, water quality, fisheries habitat, and resources is a major research and management challenge. Identification of key factors governing the ecological dynamics and environmental pollution is strongly required to establish the basis for the ecological and environment research needed to undertake the efficient management and biodiversity conservation of coastal bay ecosystems[10].

Water quality assessment encompasses monitoring, data evaluation, reporting, and dissemination of the condition of the aquatic environment. A growing number of coastal water monitoring programs is crucial to collect huge datasets. Huge datasets are important to obtain a better understanding on how anthropogenic activities have influence on the coastal water environment. The application of different multivariate approaches(cluster analysis (CA) and principal components analysis (PCA)) for the interpretation of these complex data matrices offers a better understanding of water quality and ecological status of the studied systems [4, 11]. However, few studies have applied principal component analysis coupled cluster analysis to identify anthropogenic effects on water quality.

Determination of trophic status is an important aspect of water body surveys. The objectives of this paper are: (1) to assess the temporal and spatial variations of the trophic status in Sanya, (2) and to identify key factors governing trophic status of the Sanya. It is also hoped that information generated from this study can stimulate the development of appropriate indicators and indices for trophic status along the coast of Sanya, South China Sea.

## Materials and Methods

### Study area

**Fig 1:**
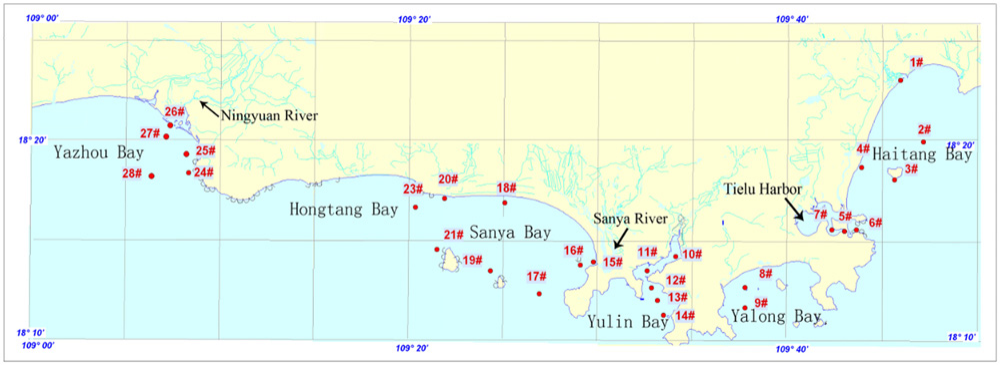
Monitoring stations along the coast of Sanya.

Sanya is situated in the southern part (from 108°56′30″ to 109°48′28″E, 18°09′34″ to 18°37′27″N) of Hainan Island, with a land area of 1919.58 km^2^ and coastline of 209.1km.There are 10 principal islands, and the largest island is Dongmao Island, Ximao Island. With the rapid development of urbanization and tourism, Sanya coastal water has experienced anthropogenic impact, receiving agricultural, domestic and industrial sewage as well as rainwater from rivers, in addition to nutrient enrichment and toxins derived from the cage culture of fishes [12]. There are 10 rivers total in Sanya city. The Ningyuan River, the longest river in Sanya, flowing into the eastern part of Yazhou bay, is 90.2 km long, drains an area of 1073.61 km^2^[13].The Sanya River, flowing into the eastern part of Sanya bay, is 31.3 km long, drains an area of 337 km^2^ and has an annual flow of 2.11×10^9^ m^3^[12]. As for the mariculture, Yulin Bay and Tielu Harbor are important inner bay aquatic areas in Sanya. The wet, warm southwest monsoon prevails in the rainy season from April to September and brings humid air from low latitudes, resulting in gentle monsoonal rainfall in spring and heavy rainfall in summer. By contrast, a dry, cold northeast monsoon predominates in the dry season from October to the following March. In order to evaluate both the anthropogenic and natural effects on the coast water in Sanya, 27 monitoring stations were located in its waters (Fig 1).

### Sampling and chemical analysis

Two cruises were carried out in the representative months of the dry and wet seasons by Sanya Marine Environmental Monitoring Station, State Oceanic Administration. During spring (representing transition period from northeast monsoon to southwest monsoon), sampling was carried out in May. During autumn (representing transition period from southwest monsoon to northeast monsoon), sampling was carried out in November in 2013.

A Quanta Water Quality Monitoring System (Hydro lab Corporation, USA) was employed to collect the data for temperature in the surface layers. Seawater samples for analysis of nutrients were taken using 5 L GO FLO bottles in the surface layers, in accordance with the methods and sampling tools of “The specialties for oceanography surveying” (GB17378.4–2007, China). Nutrients (NH_4_-N, NO_3_-N, NO_2_-N and PO_4_-P), COD, total suspended matter (TSM) and Chl-a were tested according to “The specialties for marine monitoring” (GB17378.4–1998, China). Dissolved oxygen (DO) was determined with the method of Winkler titration. Surface water was filtered through 0.45μm filter membrane and stored in a PVC bottle at 4°C prior to analysis. Heavy metals (As, and Hg) were analyzed on Atomic fluorescence photometer (PF6). Heavy metals (Cu, Zn, Pb, and Cd) were analyzed on Multifunction polarography (RC-2000D).

### Data analysis

#### One-way analysis of variance (ANOVA)

In statistics, the one-way analysis of variance tested equality of population medians among groups. In this study, ANOVA was employed to compare the temporal and spatial differences of the samples.

#### Cluster analysis (CA)

In this study, hierarchical agglomerative CA was performed on the normalized data set by means of the Ward’s method, using squared Euclidean distances as a measure of similarity [14–17].

#### Principal component analysis

PCA is designed to transform the original variables into new, uncorrelated variables (axes), called the principal components, which are linear combinations of the original variables. The new axes lie along the directions of maximum variance [6,16].

Data was auto-scaled in order to avoid misclassification due to wide differences in data dimensionality. The data were normalized with mean and variance of zero and one, respectively. Values below the detection limit were replaced by limits of detection. The Kaiser-Meyer-Olkin (KMO) was performed on the parameter correlation matrix to examine the validity of the PCA. The KMO is 0.61, and indicating that PCA may be useful in providing significant reductions in dimensionality. All the mathematical and statistical computations were performed using MATLAB R2008b (Mathworks Inc., USA).

## Results

Results of ANOVA indicated that these parameters (temperature, NO_3_-N, COD, Chl-a, As, Hg and Cd) are significant difference between May and November (Table 1).The linear correlation coefficients between the variables are shown in Table 2. TSM, inorganic nitrogen (NO_3_-N and NO_2_-N) is positively correlated with Chl-a.

**Table 1:** Descriptive statistics of water quality parameters and the one-way analysis of variance for difference between May and November.

	min		max		mean		std		Fstatistic	p value
	May	Nov	May	Nov	May	Nov	May	Nov		
Temperature										
**°C**	26.2	25.8	32.8	28.0	29.5	26.6	1.8	0.6	63.75	**0.00**
**pH**	8.04	8.10	8.25	8.22	8.14	8.16	0.05	0.04	2.05	0.16
**DO mg L** ^**-1**^	5.88	4.65	6.68	7.17	6.36	6.29	0.20	0.60	0.38	0.54
**NO**_**2**_ **-N μmol L** ^**-1**^	-	0.06	6.39	5.71	0.33	1.16	1.22	1.57	4.69	**0.03**
**NO**_**3**_ **-N μmol L** ^**-1**^	0.58	0.07	9.74	13.66	3.29	5.09	1.92	5.19	2.85	0.10
**NH**_**4**_ **-N μmol L** ^**-1**^	0.50	0.57	25.64	25.29	10.62	7.96	6.36	7.73	1.92	0.17
**DIN μmol L** ^**-1**^	1.43	1.14	33.71	40.43	13.98	14.21	8.19	13.61	0.01	0.94
**PO**_**4**_ **-P μmol L** ^**-1**^	0.02	0.10	0.75	0.52	0.19	0.26	0.16	0.11	2.93	0.09
**COD mg L** ^**-1**^	0.06	0.18	1.28	2.60	0.39	0.67	0.31	0.52	5.80	0.02
**TSM mg L** ^**-1**^	13.80	10.60	28.00	31.00	18.12	20.07	3.80	4.58	2.88	0.10
**Chl-a μg L** ^**-1**^	0.29	0.71	4.87	14.47	1.38	3.43	1.17	3.03	10.69	**0.00**
**As μg L** ^**-1**^	0.02	0.47	0.59	0.94	0.22	0.67	0.15	0.09	172.98	**0.00**
**Hg μg L** ^**-1**^	0.01	0.00	0.05	0.05	0.04	0.03	0.01	0.01	4.18	**0.05**
**Zn μg L** ^**-1**^	10.09	10.05	12.37	12.17	10.97	10.94	0.65	0.59	0.03	0.86
**Cd μg L** ^**-1**^	0.22	0.12	0.39	0.37	0.32	0.27	0.04	0.07	8.23	**0.01**
**Pb μg L** ^**-1**^	0.60	0.61	0.86	0.94	0.71	0.71	0.08	0.09	0.10	0.75
**Cu μg L** ^**-1**^	2.06	2.12	2.91	2.78	2.42	2.38	0.24	0.20	0.43	0.51

*The significance level (p<0.05) is denoted in bold. “-”denotes value below the limit of detection.

**Table 2:** Linear correlation coefficients of water quality parameters.

	Tem.	pH	DO	NO_3_-N	NO_2_-N	NH_4_-N	DIN	PO_4_-P	COD	TSM	Chl-a	As	Hg	Zn	Cd	Pb	Cu
**Temperature**	1.00																
**pH**	-0.18	1.00															
**DO**	0.03	**0.26**	1.00														
**NO**_**3**_ **-N**	-0.12	-0.25	**-0.54**	1.00													
**NO**_**2**_ **-N**	0.00	**-0.37**	**-0.51**	**0.73**	1.00												
**NH**_**4**_ **-N**	**0.29**	**-0.41**	**-0.42**	**0.40**	**0.64**	1.00											
**DIN**	0.15	**-0.43**	**-0.52**	**0.65**	**0.87**	**0.92**	1.00										
**PO**_**4**_ **-P**	0.03	-0.24	**-0.35**	**0.66**	**0.56**	**0.38**	**0.53**	1.00									
**COD**	-0.15	-0.16	**-0.29**	**0.36**	**0.60**	**0.30**	**0.46**	**0.31**	1.00								
**TSM**	-0.07	0.12	-0.11	0.09	0.14	-0.04	0.05	0.16	0.18	1.00							
**Chl-a**	-0.20	-0.19	**-0.34**	**0.54**	**0.58**	0.12	**0.37**	0.23	**0.76**	**0.28**	1.00						
**As**	**-0.68**	0.07	-0.17	0.24	**0.28**	-0.02	0.13	0.25	**0.37**	0.19	**0.35**	1.00					
**Hg**	**0.38**	-0.15	0.09	0.11	0.11	0.00	0.06	0.08	-0.16	0.12	-0.07	**-0.27**	1.00				
**Zn**	-0.03	-0.10	-0.08	-0.03	-0.01	-0.06	-0.05	-0.12	0.20	**0.42**	0.24	-0.04	0.13	1.00			
**Cd**	0.09	0.19	-0.06	-0.25	-0.25	-0.08	-0.19	**-0.33**	-0.04	-0.02	-0.08	**-0.41**	-0.06	0.23	1.00		
**Pb**	-0.06	0.06	0.25	-0.14	-0.19	**-0.36**	**-0.32**	**-0.37**	-0.12	-0.06	0.09	-0.08	0.22	**0.27**	0.00	1.00	
**Cu**	0.19	-0.05	-0.06	-0.03	0.08	0.24	0.17	0.01	-0.10	-0.10	-0.10	-0.08	0.19	0.03	0.09	-0.07	1.00

*The significance level (p<0.05) is denoted in bold.

**Fig 2:**
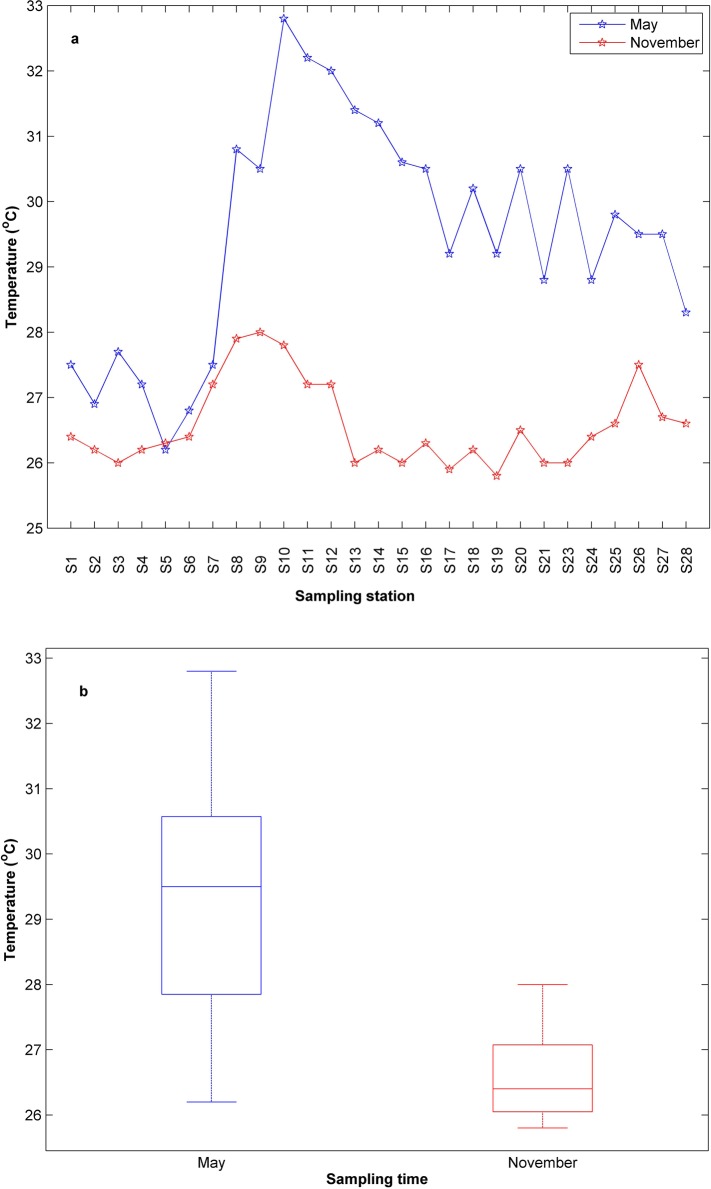
Spatial and temporal variabilities of temperature (a) and boxplot (b) along stations in the coast of Sanya.

The surface temperature exhibited a clear season variation in the bay (Fig 2a). The surface water temperature varied from25.8°C in November to 32.8°C in May. The mean sea surface temperature were 29.5°C in May and 26.6°C in November (Fig 2b). Sea surface temperature are the significant difference between the two monsoonal periods as shown through ANOVA (p = 0.00).

**Fig 3:**
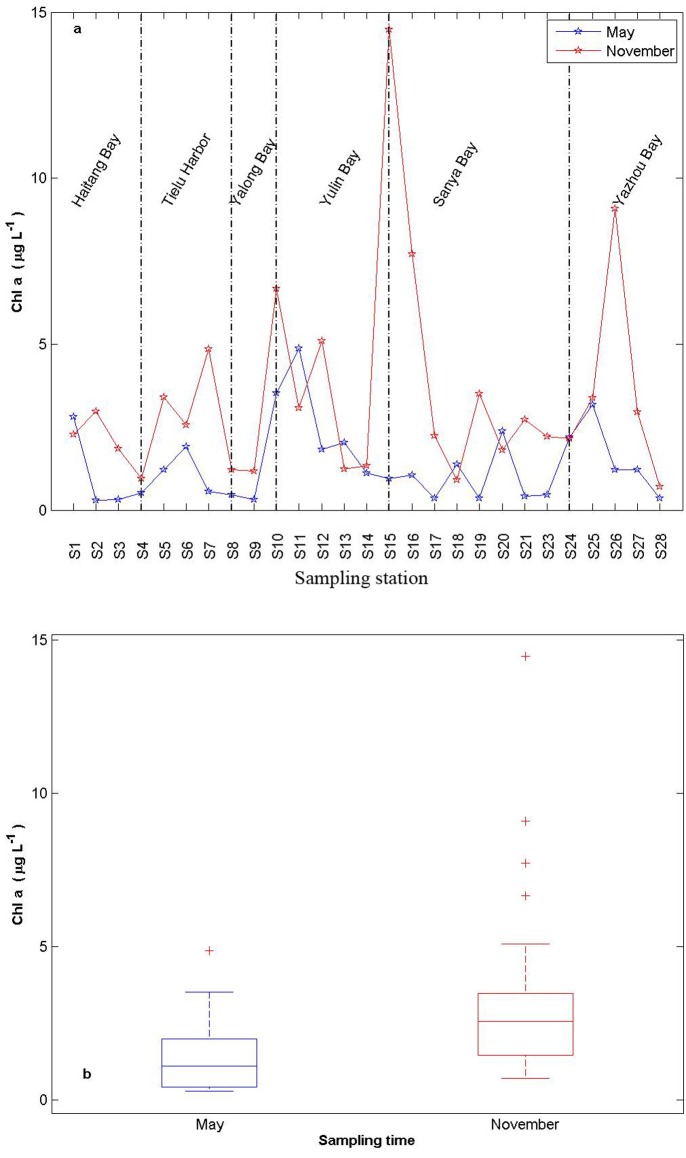
Spatial and temporal variabilities of Chl-a (a) and boxplot (b) along stations in the coast of Sanya.

Chl-a concentration ranged from 0.29 to 14.47μg L^-1^, and was higher in Sanya river and Ningyuan river estuary (S15 and S26, respectively) in November than at other monitoring stations. It is obvious that Chl-a concentration in November was higher than in May (Fig 3).

**Fig 4:**
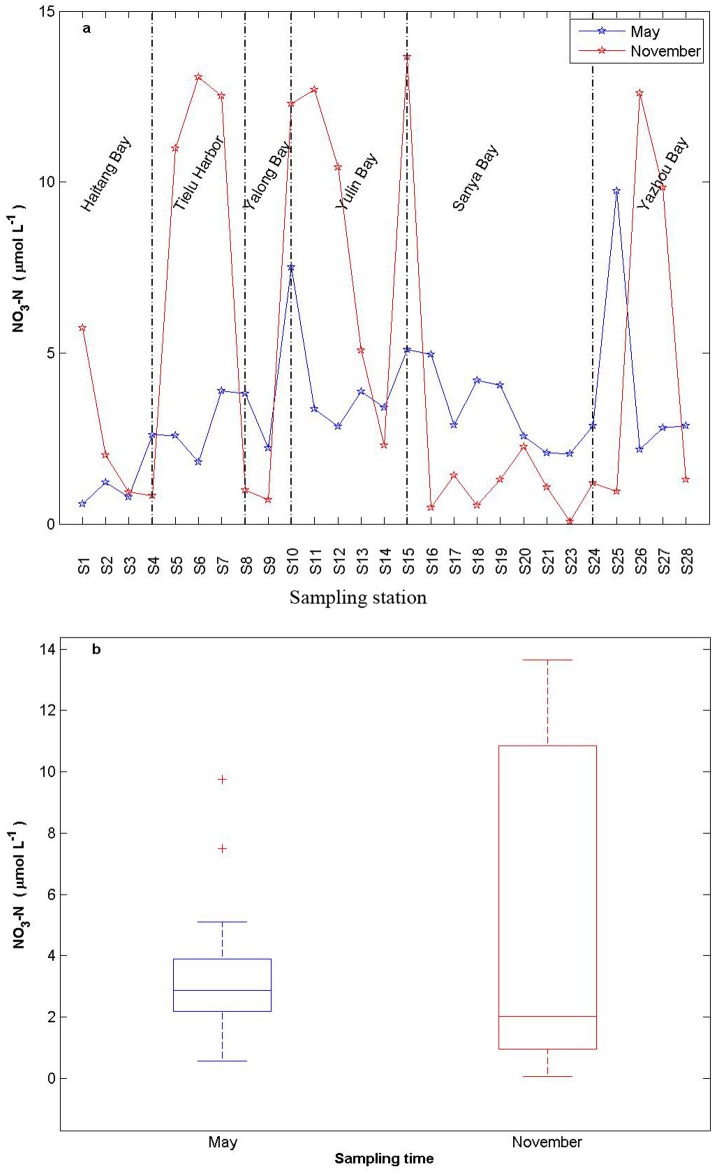
Spatial and temporal variabilities of NO_3_-N (a) and boxplot (b) along stations in the coast of Sanya.

The spatial and temporal variations in nutrient concentrations at the sampling stations were shown in Fig 4. Nitrate concentrations varied from 0.58 to 9.74μmol L^-1^ in May, and from 0.07to13.66μmol L^-1^ in November, respectively. 4 nitrate concentration peaks in November and they were in Tielu Harbor, Yulin Bay, Sanya River and Ningyuan River, respectively.

**Fig 5:**
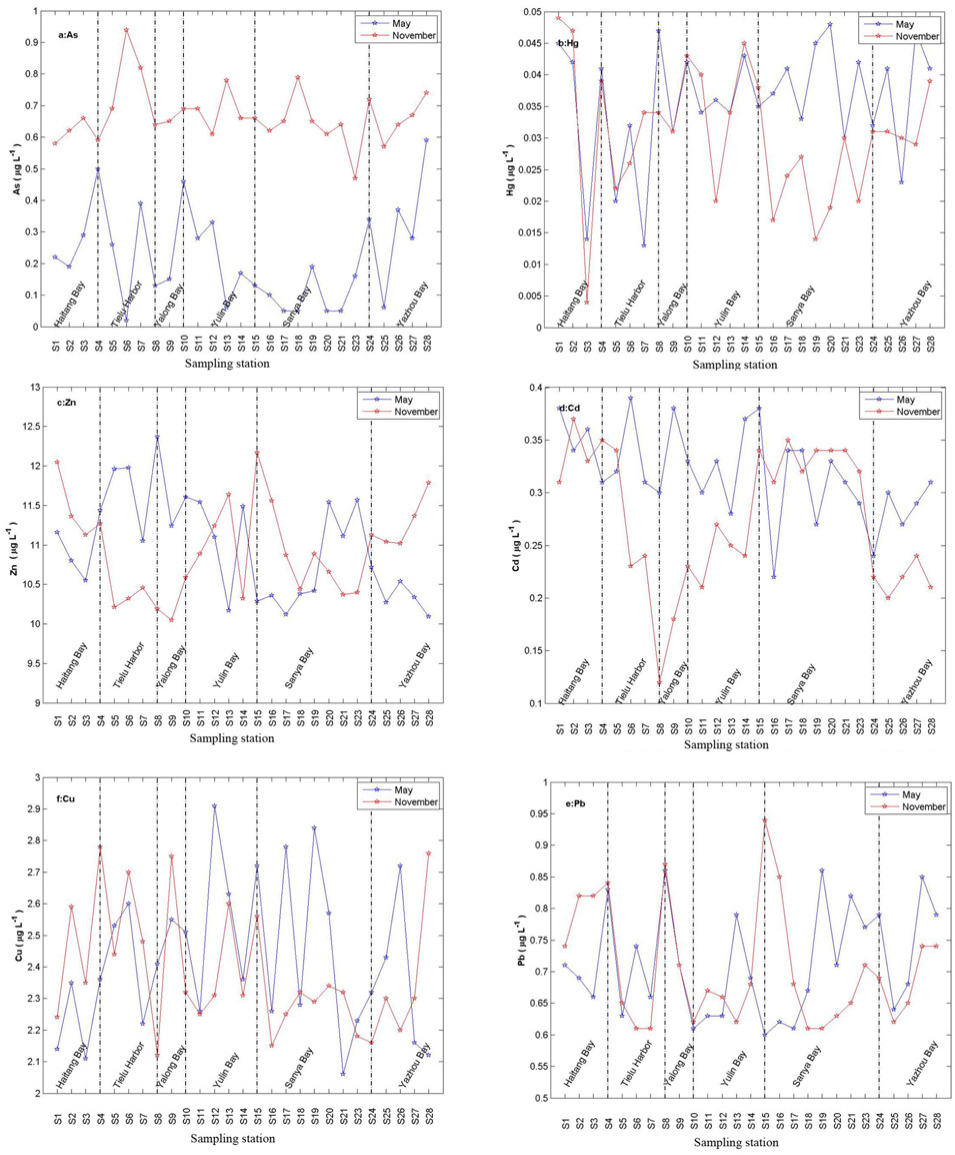
Spatial and temporal variabilities of (a) As, (b)Hg, (c)Zn, (d) Cd, (e) Pb, and (f)Cu in surface water along stations in the coast of Sanya.

The heavy metal (As,Hg and Cd) concentrations in surface water body exhibit a distinct season change (p<0.05). Seasonal change of As concentration was different from other heavy mental (Fig 5). As’ concentration in November was higher than that in May.

**Fig 6:**
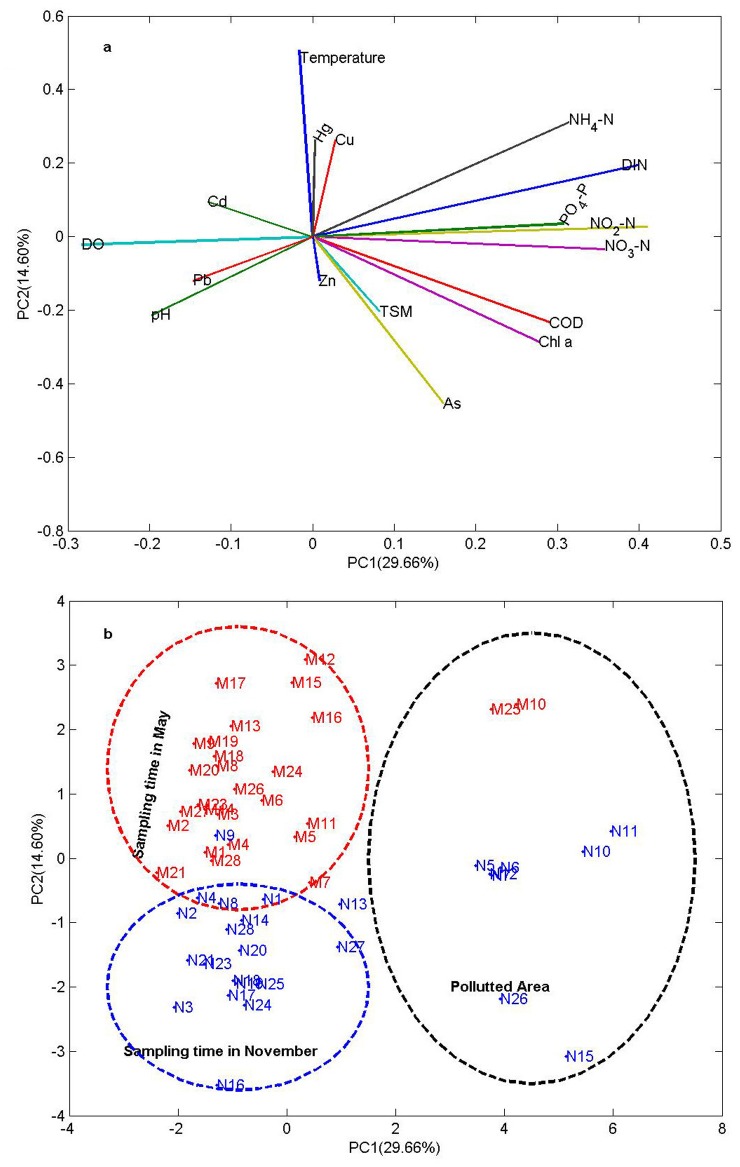
The loadings of variables (a) and scores (b) of the monitoring stations for the first two PCs, respectively. The number denotes the station number; the letter denotes the variable. The uppercase M and N denote the sampling time in May and November, respectively.

PCA and CA were employed to identify the difference of hydrochemistry between two season data (dry and wet seasons). The loading of nutrients and COD were positive on the PC1 (Fig 6a). The loadings of Pb and Cd were negative on the PC1 (Fig 6a). PC1 (31.08% of the variance) was called the “nutrients or polluted” component. The loading of temperature, Hg and Cu were positive on the PC2. PC2 (14.26% of the variance) was called the “temperature and heavy metal” component. The scores of the polluted monitoring stations stayed on the right side of the first principal component axis with high nutrients (Fig 6b). The scores of the other monitoring stations stayed on the left side of the first principal component axis with high sea surface temperature. It is interesting that the stations obtained in May located in the second quadrant, and samples in November were in the third quadrant with low sea surface temperature.

**Fig 7:**
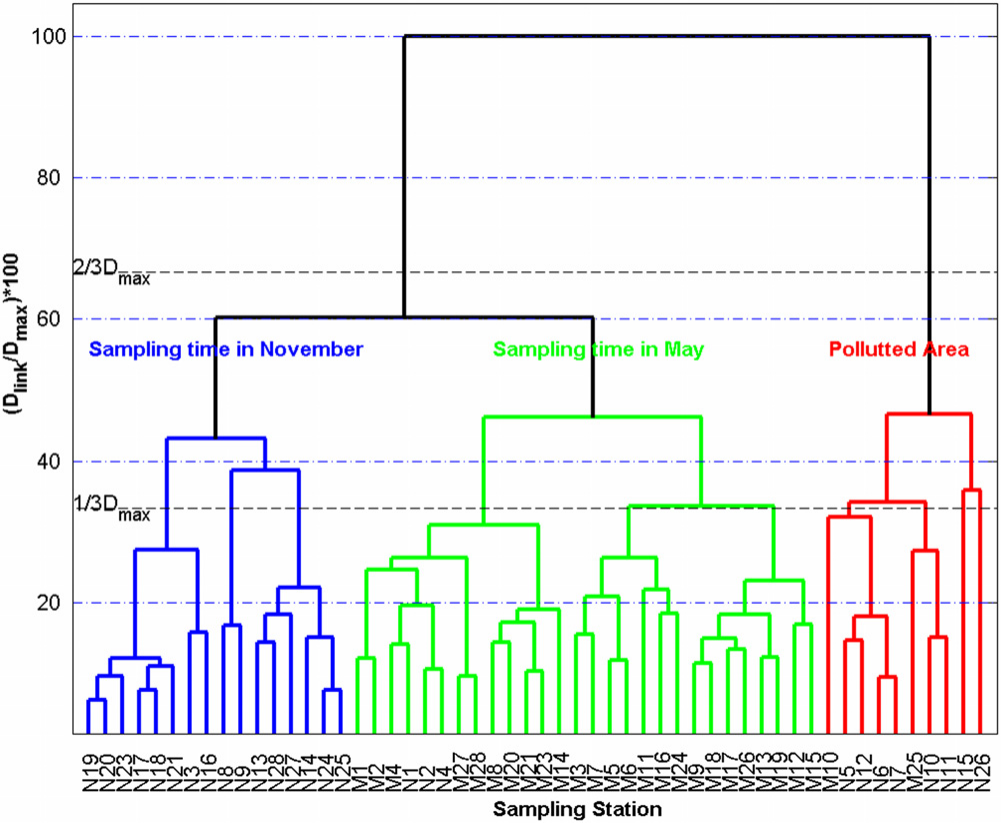
Dendrogram based on Ward’s method of clustering for 54 stations.

CA rendered a dendrogram where the monitoring stations studied were divided into three large clusters (sampling time in May, sampling time in November, and polluted area) at Dlink/Dmax*100<50 (Fig 7).

Dlink/Dmax, which represents the quotient between the linkage distances for a particular case divided by the maximal linkage distance. The quotient is then multiplied by 100 as way to standardize the linkage distance. The number denotes the station number in the horizontal axis. The uppercase M and N denote the sampling time in May and November, respectively.

## Discussion

The temporal and spatial variation of the water quality in coastal waters is governed by a variety of physical, chemical and biological processes such as advection, establishment of weather fronts, stratification, vertical mixing, water temperature, light, nutrient supply, and predation pressure [18]. It is well known that these processes are driven by human activities and nature change [4–6].

The seasonal variation in Chl-a was in the following order from the highest to the lowest: northeast monsoon (November) and southwest monsoon (May) with averages of 3.43 and 1.38μg L^-1^, respectively. The Chl-a in estuary was higher than outer bay. In this study, phytoplankton production (inferred from the seasonal change in the concentration of Chl-a) in November was higher than that in May reflecting the seasonal changes.

Temperature was insignificantly correlated with Chl-a concentration (Table 2). Temperature was not the controlling factor of the biomass, and the distribution pattern of phytoplankton in Sanya Bay[19].

Nutrient concentrations peaked in the surface waters at Sanya River mouth and Ningyuan River mouth concentrations of inorganic nitrogen nutrients (NO_3_-N, NO_2_-N and NH_4_-N) and phosphate were higher in the surface water in river estuaries. Even though discharge from Sanya River is near the sampling station (S15). Sanya River estuary with higher nutrients was in east parts of Sanya Bay, which are areas of human activity [20–22]. But, in addition to the small river flow, the Chl-a concentration was higher at S15 than at other sampling stations in November. Nutrients can stimulate phytoplankton growth in the area represented by S15. In addition, as mentioned above, nutrients were not entirely consumed by phytoplankton and remained in high concentrations at S15 located on Sanya River estuary. The supply of nutrients from river flows was low, but nutrients were relatively abundant in river estuaries [11,20]. There was remarkable increase in Chl-a levels during November (Fig 3) suggesting that these nutrients were available for phytoplankton consumption in November.

The high metal concentration (As) in the rainfall days may be attributed to inputs of freshwater runoff and suspended matter from land in November. It is known that many of the products produced in fertilizer plants, such as pesticides, insecticides and others, containing several metals (As, Hg and Cd). The first component explained 29.66% of the total variance with positive loadings on As and negative loadings on Pb and Cd (Fig 6). This component is called anthropogenic factor, which indicated that As is significantly derived from agriculture production. These elements can enter into the coastal water through surface runoff. The PCA results show that dissolved inorganic nitrogen (nitrate and nitrite), As, Cd and Pb make important contribution to PC1.

The renewal of the coastal waters along the coast of Sanya promoted by the waters of the South China Sea is an important mechanism in diluting the concentration of nutrients (eutrophication). Thus, the temporal and spatial variation of the water quality along the coast of Sanya is governed by a variety of physical, chemical and biological processes and human activities. The result of cluster analysis has demonstrated dry and wet seasons and polluted area along the coast of Sanya (Fig 7), which further supports and clarifies the seasonal pattern and polluted status. It is obviously that almost all of the stations in polluted area are near river estuaries or in aquatic areas.

## Conclusion

The status of the marine coastal waters of Sanya was assessed using principal component analysis and cluster analysis. Chl-a, nitrate and phosphate concentrations in surface layer showed a similar spatial trend, with low concentrations in outer bay and high in Sanya River mouth. PCA and CA reached the same results, and confirming the seasonality of water quality (dry and wet seasons), and polluted status (polluted area). The seasonality of water quality is related to climate and Southeast monsoons. Spatial pattern is mainly related to anthropogenic activities (especially land input). The results suggest that principal component analysis may identify the characteristics underlying the generation of coastal water quality. The temporal and spatial variation of the trophic status along the coast of Sanya is governed by a variety of physical, chemical and biological processes and human activities. The results provide a novel typological understanding of seasonal trophic status in a shallow, tropical, open marine bay.
